# Neutralizing SARS-CoV-2 Spike Antibodies against Omicron in Paired Samples after Two or Three Doses of mRNA Vaccine

**DOI:** 10.1128/spectrum.02046-22

**Published:** 2022-10-03

**Authors:** Amanda K. Debes, Shaoming Xiao, Emily R. Egbert, Patrizio Caturegli, Avinash Gadala, Elizabeth Colantuoni, Ioannis Sitaras, Andrew Pekosz, Aaron M. Milstone

**Affiliations:** a Johns Hopkins University grid.21107.35grid.471401.7 Bloomberg School of Public Health, Baltimore, Maryland, USA; b Johns Hopkins University grid.21107.35grid.471401.7 School of Medicine, Baltimore, Maryland, USA; c Johns Hopkins Health System, Baltimore, Maryland, USA; University of Georgia

**Keywords:** COVID-19, SARS-CoV-2, antibodies, omicron, protection, seroprevalence

## Abstract

SARS-CoV-2 antibody levels wane following two-doses of mRNA vaccination. An mRNA booster dose provides increased protection against hospitalization and death. We demonstrated that a booster dose provides a significant increase in the neutralization of the Beta, Delta and Omicron variants in addition to an increased neutralization of the vaccine strain. The total spike IgG measurements, obtained by using commercial kits that target the spike protein from the vaccine strain, may not reflect serum neutralization against variants of concern.

**IMPORTANCE** This study found little to no neutralizing capability following a 2-dose mRNA vaccine series against the omicron variant, and neutralizing capacity to any variant strain tested was lost by 8-months post 2-dose series. However, the mRNA booster dose eliminated the immune escape observed by the Omicron variant, following the 2-dose series. Even more, the neutralizing titers were significantly higher for all variants post-boost, compared to the titers from the post-two-dose series. Our data are unique, using paired samples that eliminate potential confounders that may impact vaccine response. Notably, as seen after the primary two-dose vaccine series, total antibody levels did not correlate perfectly with variant neutralization activity, suggesting that simply testing titers as a measure of protection may not be a long-term solution. Therefore, it is important to reassess the utility of SARS-CoV-2 antibody testing, as current vaccine strain-based testing may not reliably detect reactive antibodies to Omicron or other variants of concern.

## INTRODUCTION

SARS-CoV-2 antibody levels against the vaccine-seed strain and, even more drastically, against variants of concern (VOCs), wane as early as 3 months after the second dose of the SARS-CoV-2 mRNA vaccination series (1–3). Recent, large, population-level cohort studies suggest that the booster protects against severe disease, hospitalization, and death (4). For these reasons, the FDA approved a booster dose for people older than 12 years of age. During the recent surge, the Omicron variant’s immune evasion to many SARS-CoV-2 vaccines highlights the important need for increased booster uptake, both in the United States and worldwide. While both the primary series and booster doses protect against severe disease, the thresholds of protection in the form of antibody titers above which an individual is unlikely to experience infection or severe disease have yet to be established conclusively. The emergence of the Omicron variant encoding multiple mutations in the spike protein that affect antibody binding sites further complicates the interpretation of antibody testing data in terms of protection against and infection from severe disease. To address these questions, our objective was to use a longitudinal cohort to examine and compare total and neutralizing antibodies to the spike protein of SARS-CoV-2, as well as neutralizing antibodies against Washington-1 (WA-1) and other VOCs, including Beta, Delta, and Omicron, after the primary vaccine series and after a booster.

## RESULTS

Out of 3,032 health care workers (HWs) originally enrolled in the longitudinal cohort, 1,353 HWs contributed serum to at least one of three time points (TP) in this analysis. Of the 1,353 participants, 81% were female, 96% were non-Hispanic/Latino, and 81% were white. The median (interquartile range) age of the participants was 41.8 (33.8 to 53.3) years. Of the 1,353 HW participants, 507 were included in TP1, 879 in TP2, and 273 in TP3. Neutralizing antibody titers (NT) were performed on a subset of HWs at TP1 (*n* = 15), TP2 (*n* = 14), and TP3 (*n* = 16).

**FIG 1:**
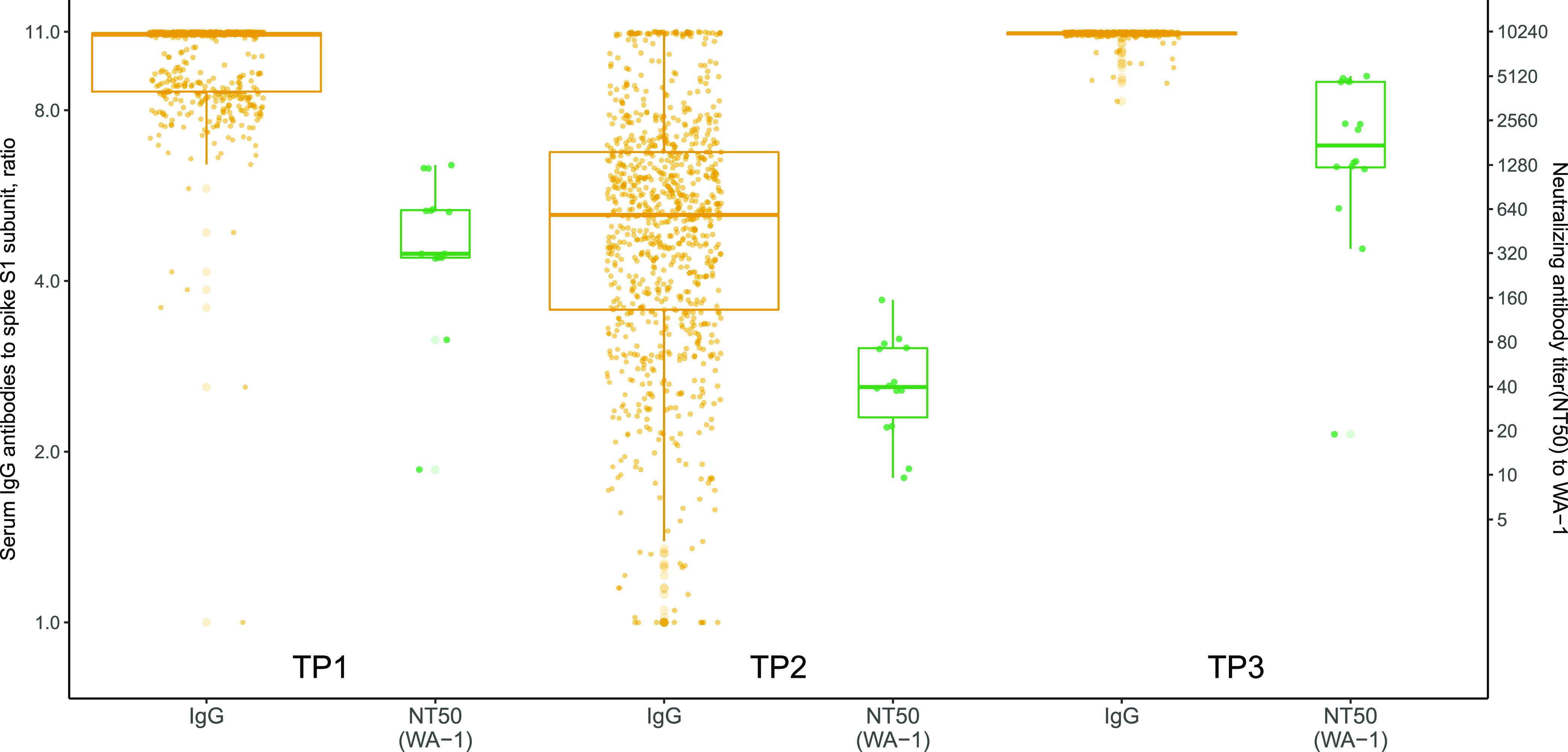
Spike IgG serum antibodies and live-virus neutralizing antibody titers (NT) against the vaccine strain (WA-1). Data are shown for three time points: within 14 to 44 days after dose 2 (time point 1), at least 8 months after dose 2 (time point 2), and within 14 to 44 post-boost (time point 3). Across the three time points, respectively: 396 (78%), 739 (84%), and 215 (79%) were women; 484 (95%), 845 (96%), and 263 (96%) were non-Hispanic/Latino; 395 (78%), 723 (82%), and 238 (87%) were white; 383 (76%), 657 (75%), 225 (82%) received Pfizer for their primary series; and the median age (interquartile range) was 39.9 (32.4, 51.9), 43.0 (35.1, 53.5), and 44.9 (34.3, 55.6).

The high levels of total spike IgG antibodies seen in TP1 waned but remained above the threshold in TP2, and then they boosted to higher values in TP3, compared to TP1 and TP2. Of the TP3 samples tested, 94% demonstrated spike IgG assay saturation, compared to 59% at TP1 (Fig. 1). Spike IgG measurements correlated with the NT antibody against WA-1, with NT antibody levels waning between TP1 and TP2 but reaching higher levels than those observed at TP1 after the boost.

**FIG 2:**
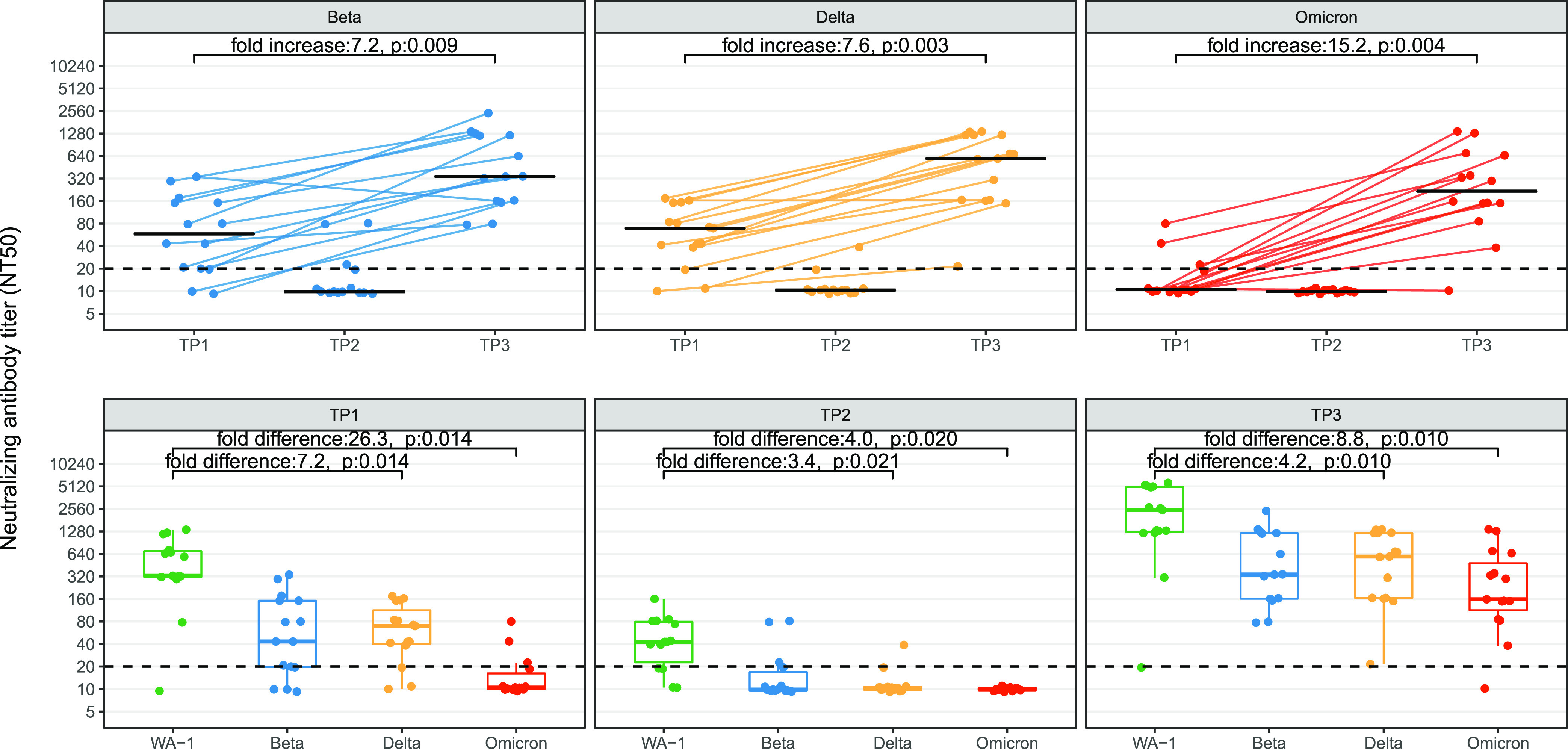
Comparison of neutralizing antibody titers (NT) to SARS-CoV-2 vaccine strain (WA-1), Beta, Delta, and Omicron VOCs from health care workers with paired serum samples in a longitudinal cohort. Data are shown for three time points: within 14 to 44 days after dose 2 (time point 1), at least 8 months after dose 2 (time point 2), and within 14 to 44 post-boost (time point 3). The top panel shows the NT titer for each variant across the three time points with connecting lines and illustrates 15 paired samples from time points 1 and 3. The bottom panel shows the NT at each time point for each VOC. The fold change (increase/difference) represents the geometric median fold change. *P* values have been corrected for multiple comparisons using the Bonferroni method.

The breadth of the NT induced by vaccination was assessed, and a similar pattern of reactivity to all three VOCs was found across the time points, with activity at TP1 waning by TP2 and then increasing following the booster at TP3 (Fig. 2, top panels). However, there were large differences in the magnitude of the NT response to specific VOCs, with WA-1 having a 7.2-fold and a 26.3-fold higher response at TP1 compared to the Delta and Omicron VOCs, respectively. The NT50s to all VOCs were nearly undetectable by TP2, but they increased to high levels at TP3, with a 4.2-fold and an 8.8-fold difference between the WA-1 NT50 and those for the Delta and Omicron VOCs, respectively, indicating an increase in the amount of cross-reactive NT antibodies following the booster (Fig. 2, bottom panels). These data suggest that the both the breadth and the magnitude of SARS-CoV-2 antibody responses are increased significantly following a booster dose.

## DISCUSSION

This study demonstrated that spike IgG antibodies and NT activity to WA-1 correlated at all three TPs, in contrast to the reduced neutralizing activity against the Beta, Delta, and Omicron VOCs that was observed at TP1 and TP2. The significant increase in cross-neutralizing activity against VOCs that was observed at TP3 further demonstrates that measuring the total spike IgG antibody levels to the vaccine strain may not reflect the ability of post-vaccination sera to neutralize SARS-CoV-2 VOCs, especially variants as antigenically distant to the vaccine seed strain as the Omicron variant (5, 6). We also demonstrate that in paired samples, an mRNA vaccine booster produces a greater quantity and function of spike antibodies and NT activity compared to primary SARS-CoV-2 mRNA immunization. This is especially so, in light of the significant antibody waning we observed prior to the introduction of the mRNA booster, which was necessary to restore measurable NT to all VOCs tested, including Omicron, which is currently the dominant circulating variant around the world (7, 8). Additionally, these results support a three-dose mRNA vaccine regimen. Further, as noted by the CDC, for a subject to be considered immunologically “up to date,” the third mRNA dose must be included in the series rather than treated as a booster. A recent publication assessing Omicron neutralization in two versus three doses of mRNA-1273 reports similar findings, including a significant waning after the second-dose and a significant increase in NT against variants after the booster. Following the third dose of mRNA-1273, the NT activity to Omicron was 20 times higher than the NT observed after the two-dose series (9).

The limitations of this study include that live-virus neutralization was only performed on a subset of the samples. Second, paired serum was only available after dose 2 and post-boost. These paired samples support prior findings that serum IgG and NT correlate against the vaccine strain (10). However, our data suggest that, given the antigenic differences between the vaccine strain and the SARS-CoV-2 variants, it is important to reassess the implications of SARS-CoV-2 antibody testing. Current vaccine strain-based testing may not reliably detect Omicron reactive antibodies and perhaps do not recognize Omicron-specific antibody responses induced by infection (11). These observations can affect policy-making decisions regarding the most appropriate type of testing for virus surveillance purposes.

Despite the correlation between the spike IgG antibodies and the neutralization of the vaccine strain, the spike IgG antibodies did not equate to protection against the VOCs tested. Although spike IgG antibody tests may be easy to obtain, and having a documented amount of SARS-CoV-2 antibodies may provide some level of reassurance for individuals. These levels do not accurately reflect the neutralizing activity or, in particular, the neutralizing activity against antigenically distant VOCs. As such, the evaluation of a vaccine’s ability to neutralize circulating SARS-CoV-2 variants should not be based on spike IgG measurements. Whether antibody measurements will provide evidence of protection from severe disease and death remains unknown. Also unknown is how long the booster immunity will last against circulating VOCs.

## MATERIALS AND METHODS

### Study population.

Health care workers were consented into a seroprevalence cohort beginning in June of 2020, and they were followed through November of 2021 (12). HWs provided serum samples longitudinally and were included in this analysis if serum was collected (i) within 14 to 44 days after the second dose of an mRNA SARS-CoV-2 vaccine (time point 1, TP1); (ii) at least 8 months after the second dose (TP2); or (iii) within 14 to 44 days following an mRNA booster (TP3). In order to evaluate vaccination-induced immune waning only, HWs with prior covid-positive PCR results were excluded from this analysis.

### Viruses and neutralization assay.

The SARS-CoV-2 viruses used were isolated by adding 150 μL of a nasal swab viral transport media (VTM) to VeroTMPRSS2 cells in a 6-well plate containing 350 μL of media as previously described (13, 14). When a complete cytopathic effect was observed, the supernatant was harvested, aliquoted, and stored at −70°C. Virus working stocks were prepared by infecting VeroTMPRSS2 cells with 150 μL of the isolate and by incubating the cells at 33°C or 37°C (the latter for the Omicron variant only). The infectious virus titers were determined via a 50% tissue culture infectious dose as previously described (15). Viruses used include an early isolate that is genetically and antigenically similar to the vaccine seed strain (hCoV-19/USA/WA1/2020), a Beta variant (SARS-CoV-2/USA/MD-HP01542/2021), a Delta variant (SARS-CoV-2/USA/MD-HP05647/2021), and an Omicron BA.1 variant (SARS-CoV-2/USA/MD-HP20874/2021). A subset of 40 five HWs were selected for analysis via neutralizing antibody assays, prioritizing paired samples from TP1 and TP3, and others were selected at random. After making 2-fold dilutions of plasma (1:20 to 1:2560), infectious virus was added at a final concentration of 1 × 10^3^ TCID50/mL to the serial dilutions, incubated for 1 h at room temperature. One hundred microliters of virus-serum mixture (containing 100 TCID50 units) was transferred to a 96-well plate of VeroE6-TMPRSS2 cells in sextuplets, and then incubated until a cytopathic effect was observed in the controls and the highest sera dilutions. The cells were fixed and stained, and the NTs were calculated as the highest serum dilutions that eliminated the cytopathic effect in 50% of the wells (NT50). A positive threshold was defined as NT ≥ 20.10.

### ELISA.

The serum at each TP was tested using an enzyme-linked immunosorbent assay (ELISA) (Euroimmun) (16) as previously described (12, 17). IgG antibody measurements were estimated based on optical density ratios with an internally derived threshold of 1.23 for greater sensitivity and specificity and an upper threshold of 11.00 based on the assay saturation (16).

### Statistical tests and analyses.

Total and neutralizing antibodies to the spike protein of SARS-CoV-2 (WA-1) and VOCs, including Beta, Delta, and Omicron were compared to determine whether the increase in the magnitude of the total antibody response to the mRNA vaccine strain led to an increased recognition and neutralization of the VOCs used. The Wilcoxon rank-sum test was used for unpaired analyses, and both the Wilcoxon signed-rank test and the Friedman test were used for paired analyses. Statistical significance is indicated by *P* < 0.01, using the Bonferroni correction for multiple comparisons. The Johns Hopkins University Institutional Review Board approved this study. Analyses were performed in R, version 4.1.2.
